# Letter to the Editor: insights into “A comparative analysis of tip-bendable suction ureteral access sheath versus traditional sheath in retrograde intrarenal stone surgery: a systematic review and meta-analysis of comparative studies”

**DOI:** 10.1097/JS9.0000000000004781

**Published:** 2026-01-13

**Authors:** Ming-Shan Lin, Wen-Juan Feng, Hui-Min Liu

**Affiliations:** a The First Clinical Medical School, Fujian University of Traditional Chinese Medicine, Fuzhou, China; b Department of Nephrology, The Third Affiliated People’s Hospital of Fujian University of Traditional Chinese Medicine, Fuzhou, China

*Dear Editor,*

We were intrigued by the systematic review and meta-analysis by Guo *et al*^[1]^, which compares the efficacy and safety of tip-bendable suction ureteral access sheaths (TBS-UAS) with traditional UAS in retrograde intrarenal stone surgery (RIRS). The authors provided valuable insights into the potential advantages of TBS-UAS, suggesting that it significantly enhances timely stone-free rates (SFRs) and reduces infectious complications, particularly in complex anatomical regions such as the lower pole. These findings have important clinical implications, potentially improving patient outcomes, enhancing surgical efficiency, and reducing post-operative complications. Such benefits are especially crucial for optimizing outcomes in RIRS, a key procedure for managing upper urinary tract stones, given persistent concerns regarding postoperative infections^[2]^. Additionally, this study lays a crucial foundation for further research on suction-assisted devices in endourology and makes an important contribution to refining surgical techniques and optimizing clinical practice.

Nevertheless, while this study represents an important contribution, we believe that addressing several key aspects in future research would strengthen the evidence base and guide clinical integration of TBS-UAS.

First, while immediate outcomes such as improved SFR and reduced complications are well-documented, long-term clinical outcomes must be thoroughly investigated^[3]^. The durability of these benefits, particularly in terms of the preservation of renal function, stone recurrence, and long-term cost-effectiveness, remains underexplored. Given that the device entails higher initial costs and a potential learning curve for clinicians, it is essential to evaluate whether the benefits of TBS-UAS are sustained over time, and to justify the associated costs and resource utilization. Future studies should include extended follow-up periods to provide a clearer understanding of the long-term impact of TBS-UAS on patient outcomes and healthcare economics.

Second, the current meta-analysis aggregated data across diverse patient populations and clinical contexts, which may have limited the assessment of TBS-UAS efficacy across subgroups. In clinical practice, the effectiveness of RIRS can vary greatly depending on stone characteristics such as size, location, density, composition, and renal anatomy, including infundibulopelvic angle and calyceal morphology^[4]^. Moreover, patient factors, including age, comorbidities, and prior stone surgery, significantly influence surgical outcomes and complication rates^[5]^. The current analysis did not fully explore these variables, which may limit the generalizability of the findings.

To address these gaps, we propose that future research prioritize individual participant data (IPD) meta-analysis. Such an approach would allow granular subgroup analyses and facilitate interaction testing among various stone, anatomical, and patient-specific variables. Therefore, future studies should identify patient populations that derive the greatest benefits from TBS-UAS. Furthermore, developing clinical prediction models that integrate variables such as stone size, location, patient age, and comorbidities could help refine patient selection and guide clinicians in making informed decisions. This would shift the research focus from establishing whether TBS-UAS is effective in determining which patients offer the most significant clinical benefits.

In conclusion, while Guo *et al*’s study offers compelling evidence in favor of TBS-UAS for enhancing the SFR and reducing complications in RIRS, further research is necessary to establish its long-term clinical efficacy and evaluate its utility across different patient subgroups. Subgroup analyses based on stone characteristics, renal anatomy, and patient factors are essential for determining which patients will benefit the most from this technology. Implementation of an IPD meta-analysis and development of clinical prediction models will provide more precise, evidence-based recommendations for the use of TBS-UAS in clinical practice. Ultimately, such efforts will help precisely identify the patient populations most suitable for TBS-UAS, thereby maximizing its clinical benefits within a personalized stone management framework.

This letter was prepared in accordance with the Transparency in the Reporting of Artificial Intelligence-TITAN guidelines^[6]^.

## Data Availability

No new data were generated or analyzed in this commentary. All information discussed is derived from previously published literature.
